# First report of *Leishmania* RNA virus 1 in *Leishmania (Viannia) braziliensis* clinical isolates from Rio de Janeiro State - Brazil

**DOI:** 10.1590/0074-02760210107

**Published:** 2022-08-22

**Authors:** Anabel Zabala-Peñafiel, Maria Fantinatti, Geovane Dias-Lopes, Jéssica Leite da Silva, Luciana de Freitas Campos Miranda, Marcelo Rosandiski Lyra, Maria Inês Fernandes Pimentel, Fátima Conceição-Silva, Carlos Roberto Alves

**Affiliations:** 1 Fundação Oswaldo Cruz-Fiocruz, Instituto Oswaldo Cruz, Laboratório de Biologia Molecular e Doenças Endêmicas, Rio de Janeiro, RJ, Brasil; 2 Fundação Oswaldo Cruz-Fiocruz, Instituto Oswaldo Cruz, Laboratório Interdisciplinar de Pesquisas Médicas, Rio de Janeiro, RJ, Brasil; 3 Fundação Oswaldo Cruz-Fiocruz, Instituto Oswaldo Cruz, Laboratório de Imunoparasitologia, Rio de Janeiro, RJ, Brasil; 4 Fundação Oswaldo Cruz-Fiocruz, Instituto Nacional de Infectologia Evandro Chagas, Laboratório de Pesquisa Clínica e Vigilância em Leishmanioses, Rio de Janeiro, RJ, Brasil

**Keywords:** *Leishmania* RNA Virus 1, Rio de Janeiro, Leishmania (Viannia) braziliensis, clinical isolates

## Abstract

**BACKGROUND:**

*Leishmania* parasites carry a double-stranded RNA virus (*Leishmania* RNA virus - LRV) that has been divided in LRV1 and LRV2.

**OBJECTIVES:**

*Leishmania (Viannia) braziliensis* clinical isolates were assessed in order to determine LRV presence.

**METHODS:**

Two-round polymerase chain reaction (PCR and nested PCR) was performed to detect LRV1 or LRV2 in *L. (V.) braziliensis* clinical isolates (n = 12).

**FINDINGS:**

LRV1 was detected in three clinical isolates which was phylogenetically related to other sequences reported from other American tegumentary leishmaniasis (ATL) endemic areas of Brazil. Patients infected with *L. (V.) braziliensis* LRV-negative showed only cutaneous lesions while LRV-positive reported different manifestations.

**MAIN CONCLUSION:**

Data presented here show for the first time that LRV1 is circulating in *L. (V.) braziliensis* clinical isolates from Rio de Janeiro State in Brazil.

American tegumentary leishmaniasis (ATL) is the term used to describe cutaneous lesions caused by *Leishmania* parasites of *Viannia* and *Leishmania* subgenres, exclusively found in the American continent.^1^ The clinical spectra of ATL is broad, including cutaneous leishmaniasis (CL), severe diffused CL, disseminated CL (DCL), metastatic and mucosal leishmaniasis (ML), and mucocutaneous leishmaniasis (MCL). Eleven species have been reported as ATL causative agents and, in Brazil, ten of them are related to ATL: *Leishmania* (*Viannia*) *braziliensis*, *L.* (*V.*) *guyanensis*, *L. (V.) panamensis*, *L.* (*V.*) *lainsoni*, *L.* (*V.*) *naiffi*, *L.* (*V.*) *shawi*, *L. (V.) utingensis*, *L. (V.) lindenbergi*, *L.* (*Leishmania*) *amazonensis* and *L.* (*L.*) *mexicana*.^2^ ^-^ ^8^ The diverse clinical manifestations of ATL vary according to *Leishmania* spp.,^9^ ^,^ ^10^ immune state of the mammalian host^8^ ^,^ ^9^ and parasite virulence factors.^11^ ^,^ ^12^

*Leishmania* parasites carry a double-stranded RNA virus (LRV) that has been divided, according to genetic distances between LRV types found on infected *Leishmania* strains, in LRV1 and LRV2.^13^ To date, LRV1 has been detected in clinical isolates from Bolivia, Colombia, Costa Rica, Ecuador, French Guiana and Peru,^14^ ^,^ ^15^ ^,^ ^16^ ^,^ ^17^ while LRV2 only in isolates from Middle eastern and African countries, showing LRV geographical distribution.^18^ ^,^ ^19^ ^,^ ^20^ Specifically, in Brazil, LRV1 has been detected in clinical isolates of *L. (V.) braziliensis*, *L. (V.) panamensis*, *L. (V.) guyanensis*, *L. (V.) lainsoni*, *L. (V.) naiffi* and *L. (L.) amazonensis* from Minas Gerais, Rondônia and Amazonas states.^17^ ^-^ ^25^ Interestingly, RNA virus in some *Leishmania* spp. is considered as a virulence factor associated with the development of severe forms of ATL. For instance, *L. (V.) guyanensis* metastatic strains had a higher rate of LRV1 positivity than non-metastatic strains and, during macrophages infection, caused over-expression of proinflammatory mediators such as TNF-α and IL-6.^26^ Furthermore, these authors also showed that macrophages treated with purified LRV1 had a similar phenotype compared to the ones infected with the metastatic strains, expressing not only higher levels of TNF-α and IL-6 but also α-chemokine and β-chemokines, which compose a typical immune profile of patients developing MCL.^26^ ^,^ ^27^ Similarly, using *L. (V.) guyanensis*, it was shown that LRV1 can be transmitted through exosomes that are secreted to the extracellular environment from multivesicular bodies and/or the parasite flagellar pocket.^28^ This is interesting since it was demonstrated, on *in vivo* models, that co-inoculation of *L. (L.) mexicana* and *L. (V.) panamensis* with their respective exosomes increased lesion size but co-inoculation with *L. (V.) guyanensis* LRV positive exosomes exacerbated lesion development.^28^ Another study revealed that LRV1 positivity frequency in *L. (V.) guyanensis* and *L. (V.) braziliensis* isolates from patients with MCL was higher than in patients with CL.^23^ However, the data on the subject is contradictory, as another study showed a similar LRV1 detection rate among *L. (V.) braziliensis*, *L. (V.) guyanensis* and *L. (V.) peruviana* metastatic and non-metastatic strains in a longitudinal cohort of ATL patients from Peru.^29^ Also, in a cohort of ATL patients from the southeast, north and northeast regions of Brazil, less than 5% of strains were LRV1 positive and the severity of the disease was related to other factors such as age, gender and immune status of the hosts.^22^ In another study with 40 *L. (V.) braziliensis* isolates from Minas Gerais State, no sample was positive for LRV1.^30^ In this context, we report for the first time that *L. (V.) braziliensis* clinical isolates from ATL patients living in Rio de Janeiro State can be infected with LRV1. In fact, the results presented here contribute to reinforce the heterogeneity previously seen for these clinical isolates.^31^ ^,^ ^32^

## MATERIALS AND METHODS

In this study, RNA was obtained from stationary-phase promastigotes (10^7^ to 10^8^ parasites/mL) of *L. (V.) braziliensis* clinical isolates (n = 12) and positive control sample [IOC/L0565 (MHOM/BR/1975/M4147) *L. (V.) guyanensis*], cultured *in vitro* as previously described.^31^ Each sample was lysed in TRIzol containing chloroform, and RNA was extracted using RNeasy Mini Kit (QIAGEN, Germany). Then, RNA samples were converted into cDNA, using High-Capacity cDNA Reverse Transcription kit (Applied Biosystems, USA), to detect LRV1 and LRV2 with specific *primers* in a two-round polymerase chain reaction (PCR) (Table I). Both PCR and nested PCR were conducted in a final volume of 25 µL of reaction containing 1X PCR buffer, 3 mM of MgCl_2_, 2.5 U of Taq DNA Polymerase (Invitrogen Life Technologies, Brazil), 200 mM of triphosphate deoxyribonucleotides dNTP (Invitrogen Life Technologies, Brazil) and 0.2 µM of each primer, and the PCR assay conditions were performed as described in Table I. The amplicons obtained from nested PCR were purified using the NucleoSpin^®^ Gel and a PCR Clean-up kit (Macherey-Nagel GmbH & Co. KG, Germany), with a minor change in incubation (increased to five minutes). The purified products were subjected to sequencing in both directions in triplicate using the ABI Prism™ BigDye Terminator Cycle Sequencing kit (Applied Biosystems, USA) on an ABI 3730 automatic DNA sequencer at Fiocruz facilities [Capillary Electrophoresis DNA Sequencing Platform (SANGER) - RPT01A].^33^ The obtained sequences were deposited in GenBank under accession number ON409677-ON409679. Electropherograms were analysed using Chromas 2.4, while percent identity with sequences producing significant alignments was performed using the Basic Local Alignment Search Tool using nucleotide (BLASTn). Nucleotide sequences were aligned by the CLUSTAL W algorithm from Molecular Evolutionary Genetics Analysis (MEGA) X.^34^ The phylogenetic analysis was performed using MEGA and the range estimation equations used were JIN and NEI (Kimura 2-parameter model). For the LRV positive samples, previously characterised *Leishmania Viannia* species were also confirmed by SANGER sequencing using *HSP70* gene.^35^

**TABLE I:** Polymerase chain reaction (PCR) assay conditions for detection of *Leishmania* RNA virus (LRV)

		Name	Sequence (5’→3’)	Denaturation	Annealing	Extension	Cycles	Amplicon size (bp)	References
LRV1	PCR	P1-LRV1-Fw P1-LRV1-Rev	CTGACTGGACGGGGGGTAAT CAAAACACTCCCTTACGC	95ºC/30s	55ºC/30s	72ºC/30s	35	124	19
	Nested PCR	P2-LRV1-Fw P2-LRV1-Rev	GGTAATCGAGTGGGAGTCC GCGGCAGTAACCTGG	95ºC/30s	55ºC/30s	72ºC/30s	35	90	19
LRV2	PCR	P1-LRV2-Fw P1-LRV2-Rev	TGTAACCCACATAAACAGTGTGC ATTTCATCCAGCTTGACTGGG	95ºC/10s	60ºC/1min	72ºC/30s	35	526	31
	Nested PCR	P2-LRV1-Fw P2-LRV1-Rev	AGGACAATCCAATAGGTCGTGT ATTTCATCCAGCTTGACTGGG	95ºC/35s	60ºC/35s	72ºC/45s	35	315	31

## RESULTS

**Fig. 1:**
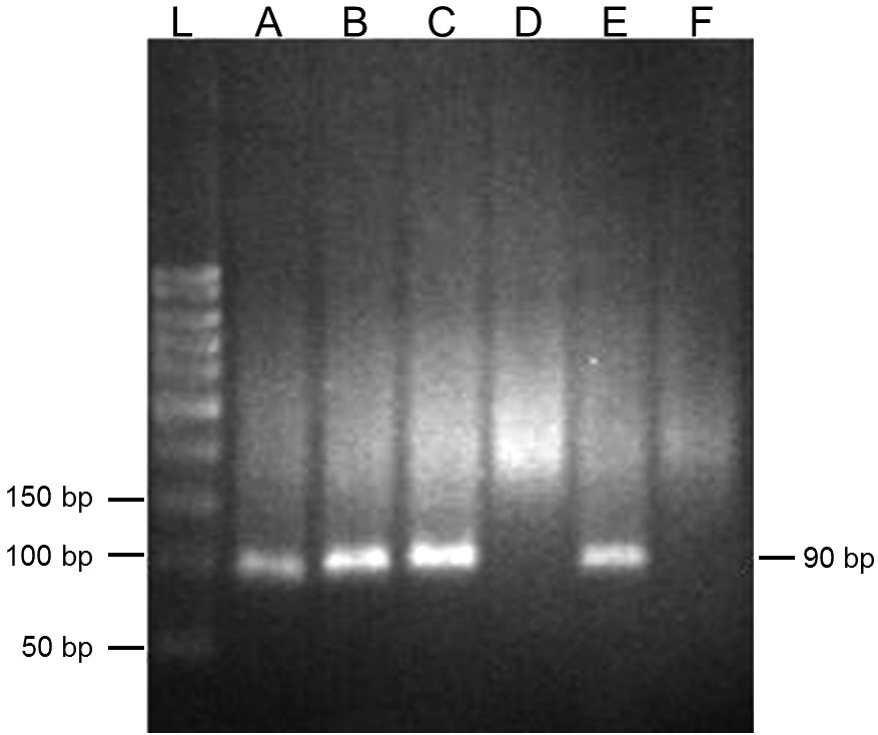
identification of *Leishmania* RNA virus (LRV) in *L. (V.) braziliensis* clinical isolates in electrophoresis in agarose gel 3%. L: molecular size marker (50 pb) A, B and C: LRV1 from isolates 7, 11 and 12, respectively. D: negative control (*Leishmania* without LRV). E: positive control (*Leishmania* with LRV). F: only polymerase chain reaction (PCR) mix.

**Fig. 2:**
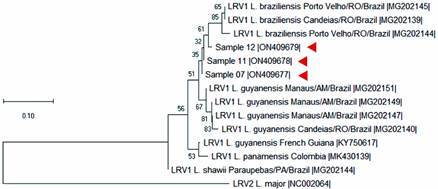
phylogenetic tree of the *Leishmania* RNA virus in *L. (V.) braziliensis* clinical isolates collected from patients living in Rio de Janeiro-Brazil, by the neighbor-joining algorithm using a Kimura two-parameter. Red arrows indicate the position of the samples of this study. Phylogenetic trees were constructed using the neighbor-joining algorithm, with bootstrap analysis (1000 replicates). The sequence from the new isolates were aligned using sequences of LRV from GenBank (KY750617, MG202139, MG202140, MG202142, MG202144, MG202145, MG202147, MG202149, MG202151, MK430139, NC0022064).

The presence of LRV1 was identified in three *L. (V.) braziliensis* isolates from patients living in Rio de Janeiro by gel electrophoresis (Fig. 1) and confirmed by sequencing. Phylogenetic analysis indicates that the LRV1 have greater identity compared to other LRV1 identified in *L. (V.) braziliensis* isolated from patients of other Brazilian cities, such as Porto Velho and Candeias of Rondonia State (Fig. 2).

## DISCUSSION

In accordance with LRV reported by other authors in Latin America, LRV2 was not identified in this study. Although all the individuals were residents of Rio de Janeiro, one of the patients was infected by *L. (V.) braziliensis* presumably in the municipality of Carangola in Minas Gerais State (Table II), where the presence of LRV has been previously reported in patients with CL.^36^ The infection by *L. (V.) braziliensis* of the other two patients in the study occurred in the state of Rio de Janeiro (municipalities of Barra Mansa and Rio de Janeiro) (Table II), where circulation of LRV has not been reported so far. In addition, to confirming LRV1, it was possible to observe the following single-nucleotide polymorphisms among the isolates: T/A (position 35, isolates 7 and 11 versus 12), T/G (position 37, isolates 7 and 11 versus 12), T/C (position 65 isolates 7 and 12 versus 11), G/A (position 71, isolates 7 and 12 versus 11). Moreover, it is important to mention that these three clinical cases have a more exuberant disease profile, and at least two needed subsequent treatments, while all the negative cases are associated to patients with CL (Table II).

In conclusion, the findings presented here are original and serve as an alert that LRV1 is circulating in *L. (V.) braziliensis* in Rio de Janeiro. Additional studies with more clinical isolates are needed to assess a possible correlation between LRV1-infected parasites, clinical manifestations and treatment response, as described for ATL in other endemic areas.

**TABLE II:** Clinical characteristics of patients infected with *Leishmania (Viannia) braziliensis*

Parasite isolate	Age/Sex	Occupation	Local of infection*	Clinical manifestation	Clinical response**	Subsequent treatments***	LRV1	LRV2
1	41/M	Farmer	Campo Grande/RJ/RJ	CL	NR	Meglumine antimoniate (1 round)	-	-
2	59/M	Garbage recycler	Campo Grande/RJ/RJ	CL	NR	Meglumine antimoniate (3 rounds)	-	-
3	31/M	Military	-/Manaus/AZ	CL	NR	Meglumine antimoniate (1 round)	-	-
4	24/M	Self employed	Mazomba/Itaguaí/RJ	CL	R	NST	-	-
5	55/F	Nurse	Santíssimo/RJ/RJ	CL	R	NST	-	-
6	48/M	Housekeeper	Cidade Jardim Marajoara/Japeri/RJ	CL	R	NST	-	-
7	48/M	Self-employed	-/Barra Mansa/RJ	DCL 24 lesions	NR	Meglumine antimoniate (1 round) Amphotericin B lipid complex (1 round)	+	-
8	25/F	Housewife	Caçador/Itaguaí/RJ	CL	NR	Meglumine antimoniate (1 round) Amphotericin B deoxycholate (1 round)	-	-
9	38/M	Civil construction	Campo Grande/RJ/RJ	CL	R	NST	-	-
10	52/F	Housekeeper	Itaipava/Petrópolis/RJ	CL	NR	Meglumine antimoniate (1 round) Amphotericin B deoxycholate (1 round) Pentamidine isethionate (1 round)	-	-
11	35/M	Civil construction	-/Carangola/MG	ML nose	R	NST	+	-
12	21/F	Student	Campo Grande/RJ/RJ	CL	NR	Meglumine antimoniate (4 rounds) Amphotericin B deoxycholate (1 round)	+	-

As labelled in{xe “citationID”}^31^; *neighborhood/city/state; **response after 1 round of intramuscular Meglumine antimoniate 5 mg/kg/day for 30 days until clinical cure (epithelisation); ***all patients were first treated with intramuscular Meglumine antimoniate 5 mg/kg/day for 30 days until reaching clinical cure. Who did not respond was submitted to subsequent treatments until reaching clinical cure, Meglumine antimoniate (same dose) and/or Amphotericin B (lipid complex: total dose of 1800 mg; deoxycholate: total dose of 1000 mg). AZ: Amazonas; CL: cutaneous leishmaniasis; DCL: disseminated cutaneous leishmaniasis; F: feminine; LRV1: *Leishmania* RNA virus 1; LRV2: *Leishmania* RNA virus 2; M: masculine; MG: Minas Gerais; ML: mucosal leishmaniasis; NR: non-responder; NST: no subsequent treatments; R: responder; RJ: Rio de Janeiro.
